# The Pro12Ala Polymorphism in the Peroxisome Proliferator-Activated Receptor Gamma-2 Gene (*PPARγ2*) Is Associated with Increased Risk of Coronary Artery Disease: A Meta-Analysis

**DOI:** 10.1371/journal.pone.0053105

**Published:** 2012-12-31

**Authors:** Zhijun Wu, Yuqing Lou, Wei Jin, Yan Liu, Lin Lu, Guoping Lu

**Affiliations:** 1 Department of Cardiology, Ruijin Hospital, Shanghai Jiao Tong University School of Medicine, Shanghai, China; 2 Department of Pulmonary, Shanghai Chest Hospital, Shanghai Jiao Tong University, Shanghai, China; University of Freiburg, Germany

## Abstract

**Background:**

Contradictory results have been reported regarding the association between Pro12Ala polymorphism of *PPARγ2* and coronary artery disease (CAD). We sought to estimate the inconsistent results by performing a comprehensive meta-analysis.

**Methods:**

Studies in English or Chinese publications were identified by screening MEDLINE, Embase, CNKI, Wanfang and CBM. 22 studies including 8948 cases and 14427 controls were selected. A random-effects model was applied to combine the divergent outcomes of the individual studies, while addressing between-study heterogeneity and publication bias.

**Results:**

The Pro12Ala polymorphism of control population followed Hardy-Weinberg equilibrium for all studies (P>0.05). Overall, a marginal increased risk of CAD under the recessive genetic model (AlaAla vs ProAla+ProPro: P = 0.04, OR = 1.31, 95%CI 1.01–1.69, P_heterogeneity_ = 0.67, I^2^ = 0%) and the homozygote comparison (AlaAla vs ProPro: P = 0.04,OR = 1.30, 95%CI 1.01–1.68, P_heterogeneity_ = 0.68, I^2^ = 0%) was observed. In the subgroup analysis by ethnicity, carriers of AlaAla homozygotes had a significant increased risk for CAD among Caucasians (AlaAla vs ProAla+ProPro: P = 0.01, OR = 1.45, 95%CI 1.08–1.96, P_heterogeneity_ = 0.48, I^2^ = 0%; AlaAla vs ProPro: P = 0.02,OR = 1.44, 95%CI 1.07–1.93, P_heterogeneity_ = 0.46, I^2^ = 0%). After dividing into population source, the CAD risk magnitude of hospital-based studies was distinctly strengthened under the recessive model (P = 0.03,OR = 1.85,95%CI 1.07–3.19, P_heterogeneity_ = 0.87,I^2^ = 0%) and the homozygote comparison (P = 0.03,OR = 1.83, 95%CI 1.06–3.16, P_heterogeneity_ = 0.88, I^2^ = 0%). There was no observable publication bias as reflected by funnel plot and Egger’s linear regression test (t = -0.12, P = 0.91).

**Conclusion:**

Our results demonstrated that the *PPARγ2* Pro12Ala polymorphism might be risk-conferring locus for the progression of CAD among Caucasians, but not among Asians.

## Introduction

Coronary artery disease (CAD) and myocardial infarction (MI), the leading causes of morbidity and death in industrialized countries, represent heavy economic and social burdens on the public health system. CAD and MI are common disorders resulted from the interaction of numerous risk factors [1],including Diabetes,obesity, hypercholesterolemia, hypertension, smoke and so on. In the past few years, large quantities of evidences have documented that the genetic factors may contribute to the majority of variation in susceptible to CAD. Nevertheless, little crucial genetic variants that determined the progression of CAD were found out.

The gene peroxisome proliferator-activated receptor γ (*PPARγ*), located on chromosome 3p25, is a member of the nuclear receptor superfamily. PPARγ is considered as a “master regulator” in the course of glucose homeostasis, lipoprotein metabolism and vascular homeostasis [2], [3]. PPARγ has two isoforms (γ1 and γ2), which differ at their N terminus [4]. The PPARγ2 isoform is mostly expressed in adipose tissue [5]. The most common gene polymorphism in human *PPARγ2* gene is cytosine-guanine exchange in exon B (codon12) which results proline to alanine (Pro12Ala) substitution in the protein [6].The Pro12Ala polymorphism was first identified by Yen *et al.* [7] in 1997 and regarded to reduce transcriptional activity of PPARγ2 [8], resulting in lower transcription levels of target genes [9],including tumor necrosis factor α (*TNF α*), leptin, resistin, adiponectin, and plasminogen activator in hibitor-1(*PAI*-*1*), which play important roles in the process of inflammation and atherosclerosis. There are evidences that the resulting mutant transcription factor profoundly affects the energy metabolism and energy balance and is associated with the risks of atherogenesis [10]–[12] and diabetes [13], [14]. In addition, the Pro12Ala polymorphism was regarded to change the response of synthetic PPARγ agonists– thiazolidinediones (TZDs) treatment [15], which seemed to improve the insulin resistance [16] and limit atherosclerosis development [17]. Many studies have been performed to explore the association between *PPARγ2* Pro12Ala polymorphism and CAD, but data are inconsistent [18]–[23]. As a matter of fact, single studies with restrictive sample sizes have insufficient statistical powers to determine the common variants with moderate effects on CAD and the results are not replicated most of time. Given the limitation of these individual researches, large meta-analysis is a feasible strategy to reliably assess the predetermined candidates in genetic-related researches. To derive a more precise estimation, we performed a meta- analysis of published studies to date in order to evaluate the association of *PPARγ2* gene Pro12Ala polymorphism with CAD, while addressing between-study heterogeneity and publication bias.

## Materials and Methods

### Search Strategy

To identify all studies that examined the relationship between *PPARγ2* Pro12Ala polymorphism and CAD, a systematic computerized literature search was conducted. The search was done on August, 2012. All published studies were found with PubMed/MEDLINE, Embase, CNKI (China Nation Knowledge Infrastructure Platform), Wanfang, CBM (China Biological Medicine Database) electronic databases by using the following combinations of text search string: ‘Pro12Ala’, ‘Peroxisome proliferator -activated receptor gamma’ and ‘coronary’ or ‘CAD’ or ‘myocardial infarction’ or ‘MI’. We also retrieved additional studies through the MEDLINE option ‘related articles’ and manual bibliography review was added. References from the retrieved articles, reviews, and previous meta-analysis were also screened to complete the data bank. If the data were incomplete in an appropriate format, we connected to the corresponding author to obtain the data. The following constraints were applied to the search: (1) Articles published in English or Chinese journals or their supplements; (2) Studies in human subjects without country restrictions; (3) When studies from the same research group with overlapped population were found, only the one with largest population was included to avoid data duplication; (4) Have available genotype frequency; (5) If articles containing more than one geographic or ethnic heterogeneous group, each group was treated separately; (6) genotype distribution of control population must be consistent with Hardy-Weinberg equilibrium (HWE).

CAD was defined as documented evidence of a previous MI, coronary bypass operation or coronary catheterization findings of significant stenosis of 50% or more in at least one major coronary artery together with clinical symptoms of angina [24]. Acute coronary syndromes (ACS) included unstable angina pectoris, fatal and non-fatal MI [25] and MI was defined as the presence of typical electrocardiographic changes and elevation in the levels of cardiac enzymes [26].

### Data Extraction

With the purpose of extracting the necessary characteristics, all relevant articles were collated independently and entered into separate databases by two investigators (ZW and Y. Lou). They checked for any encountered discrepancies and reached a consensus. The following information was collected on the genotype of *Pro12Ala* according to different cohort: First author’s name, publication year, geographic location and population ethnicity, study design, population source, diagnostic criteria, baseline characteristics of the study population (such as age, gender, and body mass index [BMI]), the proportion of diabetes and smoking, the Pro12Ala genotype frequency in patients and controls, genotyping methods and consistency of genotype frequencies with Hardy-Weinberg equilibrium (HWE). Quantitative variables expressed as mean ± standard deviation (SD) or median (5^th^ and 95^th^ percentiles).

### Quality Score Assessment

The quality of studies was also separately assessed by the same two investigators. Quality scoring criteria were modified from the genetic association study by Thakkinstian et al [27]. Total scores ranged from 0 (worst) to 13 (best). The criteria of quality assessment for the association of the Pro12Ala polymorphism and CAD were described in Table S1.

### Statistical Analysis

We examined the extent of the association between the *PPARγ2* pro12ala polymorphism and CAD risk by calculating odd ratio (OR) with 95% confidence interval (CI). The result of allele comparison (Ala vs Pro), the dominant genetic model (ProAla+AlaAla vs ProPro), the recessive genetic model (AlaAla vs ProAla+ProPro), and homozygote comparison (AlaAla vs ProPro) were obtained through assessing the pooled studies’ ORs. The random-effects model using the DerSimonian & Laired method was applied to calculate individual effect size together and the Mantel-Haenszel model [28] was used to evaluate the heterogeneity of the studies. The random-effects method adjusted the study weights according to the in-study variance. We assessed the between-study heterogeneity in approach to a Chi-square-based Q statistic test [29]. P<0.10 was considered significantly heterogenetic among the studies. The inconsistency index I^2^ statistic (ranging from 0 to 100%) was also documented to estimate the degree of heterogenetic variation [30], with higher values suggesting the variability of between-study was caused by heterogeneity rather than chance. The significance of the pooled OR was determined by the Z test and P<0.05 was considered to be significant. Initially studies were categorized into subgroups based on ethnicity. Three subgroups (Asian, Caucasian and others) according to different descent were analyzed for ethnic-specific genetic comparison. The population of Indian was grouped as “others” since its lineage was complicated and cannot simply be grouped as Asian or Caucasian [31]–[34]. The Costa Rica population was of Mestizo background and also grouped as “others” [35]. Pischon *et al.* [22] and Dallongeville *et al.* [36] had provided data from two different studies respectively (Nurses’ Health Study [NHS] and Health Professionals Follow-up Study [HPFS] by Pischon *et al.*; Prospective Study of Myocardial Infarction [PRIME] and Atherosclerotic Disease, Vascular Function, and Genetic Epidemiology [ADVANCE] by Dallongeville *et al.*). Although the predominant ethnicity of these studies was Caucasian, these four studies were performed in different geographical population (The PRIME study was based in France and in Northern Ireland; The patients of the ADVANCE study came from different countries globally; The NHS study and the HPFS study recruited US participates) and in distinct periods (PRIME in 1991, ADVANCE in 2001, NHS in 1976 and HPFS in 1986). Considering the heterogeneity of environment exposures of the different region and era, we regarded these studies as four independent studies in our meta-analysis. Then we estimated the other study characteristics that could classify the studies into subgroups with homogeneous effects, such as study design, population source and endpoints.

Cumulative-analysis was performed to determine the impact of the first published research on the subsequent publication and the evolution of the pooled estimates over time in accordance with the ascending date of published articles.

Sensitivity analysis was conducted by sequential deleting a single study each time in an attempt to identify the potential influence of the individual data set to the pooled ORs. Furthermore, meta-regression was used as an extension to random-effects meta-analysis. In addition, we used the funnel plot to estimate potential publication bias. The standard error of log (OR) of each study was plotted against its OR. An asymmetric plot suggested publication bias probably. Egger’s linear regression test [37] was examined to verify Funnel-plot asymmetry. We also performed a T-test to determine the significance of intercept and P<0.05 of I^2^ statistic and Egger’s test was considered to be statistically significant. HWE was tested by the chi-square test or Fisher's exact test for goodness of fit based on a Web program (http://ihg2.helmholtz-muenchen.de/cgi-bin/hw/hwa1.pl). Review Manager software release 5.0 (Oxford, England) and Stata 11.0 (Stata Corporation, College Station, Texas, USA) were used for combined data in all studies, and all P values were 2-sided.

## Result

### Description of Studies Search Result

**Figure 1:**
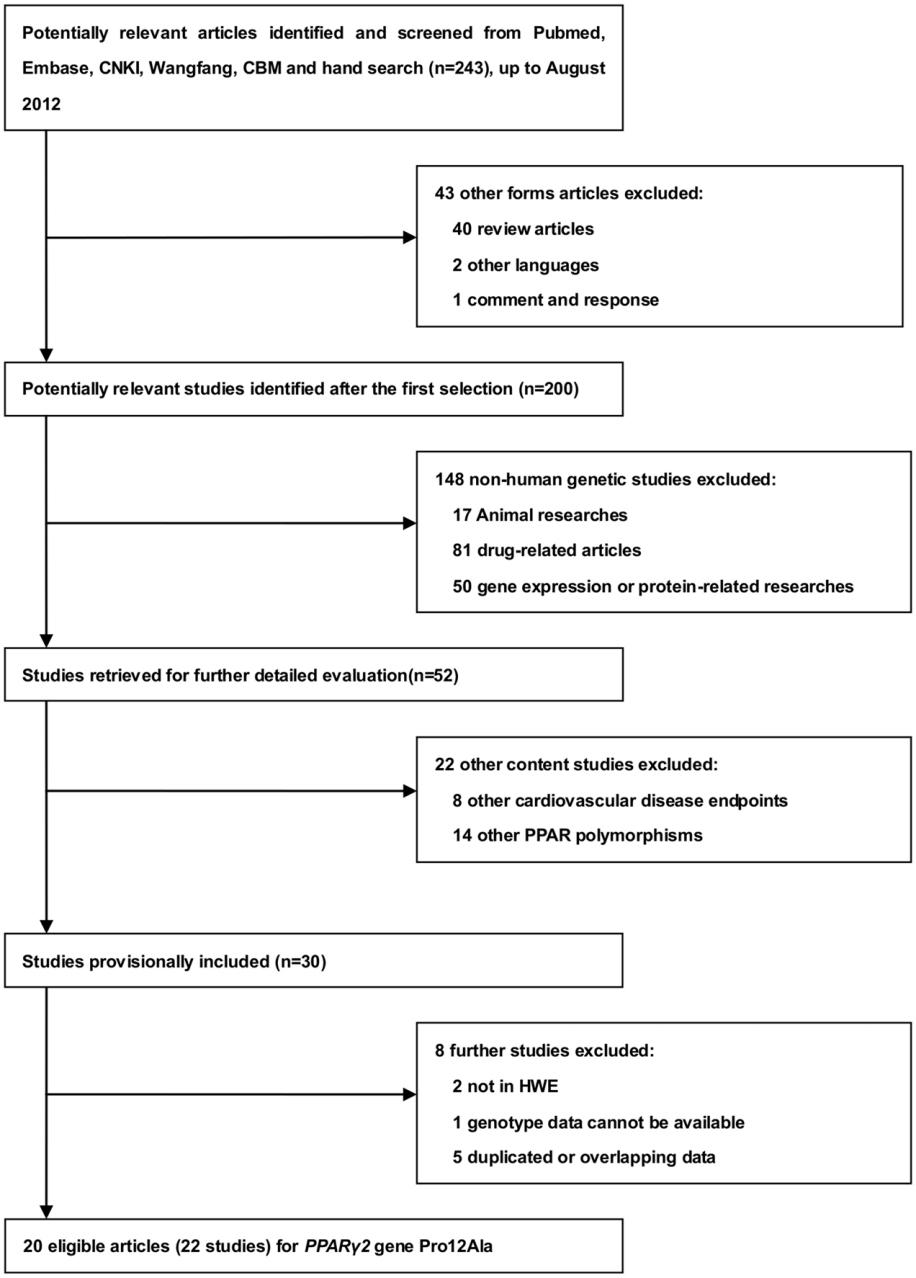
Flow diagram of search strategy and study selection for the meta-analysis.

The flowchart summarizing the process of study search and selection was presented in Figure 1. After initial literature search in PubMed, EMBASE, CNKI, Wangfang and CBM with our search strategy and manual bibliography review, a total of 243 relevant articles were yielded. After the subsequent selection, 30 studies focusing on the relationship between *PPARγ2* Pro12Ala polymorphism and CAD was provisionally included. Among these studies, 8 articles were further excluded: 2 study [19], [38] and 3 abstracts [39]–[41] were overlapped by other 3 studies [42]–[44] with larger population. The genotyping data in the controls of Wang *et al.* [45] and Galgani *et al.* [46] was deviated from HWE in control population (P_HWE_ = 8.865×10^−7^ & 0.044). These two articles were excluded. Two studies had insufficient data, so we tried to connect with the corresponding or original authors for detailed data by E mail. Until recently, the raw data by Ho JS *et al.* [43] were provided by the original author and Doney *et al.* [20] did not reply. The result of our quality score assessment varied between 8 and 12, suggesting that all the studies contained in our meta-analysis were of medium or high quality.

20 articles included 22 studies with sufficient information were identified in the light of the inclusion criteria [18], [21]–[23], [36], [42]–[44], [47]–[54]. All the eligible studies were published between 2000 and 2012, with 7 in Asian, 13 in Caucasian and 2 in others (Costa Rican & Indian). 8 of the 22 qualified studies were prospective [22], [36], [43], [44], [53], [54] and the others were retrospective. 11 studies were population-based (P–B) [22], [36], [44], [47], [49], [50], [52]–[54] and the rest half were hospital-based (H–B). 15 studies were analyzed for CAD [22], [36], [42], [43], [47], [48], [50], [51], [54] as the primary outcome, 2 studies for ACS [49], [53] and 5 studies for MI [18], [21], [23], [44], [52] as an end point.

### Overall Analysis

22 studies comprising 8948 cases and 14427 controls were selected for the meta-analysis. The baseline characteristics of the qualified studies were summarized in Table 1. The distribution of *PPARγ2* Pro12Ala genotypes and alleles in the individual studies was listed in Table 2. Genotype distribution of the Pro12Ala polymorphism of control population were in line with HWE for all eligible studies (P>0.05). The pooled overall frequency of the Ala allele was 10.6% in cases and 10.1% in controls. The highest frequency of Ala allele was observed in Caucasian population (11.6% cases vs 12.1% controls). The frequency among Asians (5.2% cases vs 3.6% controls) was much lower than that among Caucasians and others of mixed origin (10.4% cases vs 9.5% controls).

**Table 1:** The baseline charcteristics of all eligible studies in the meta-analysis.

First Author	Year	Ethnicity	geographiclocation	Design	Source	Endpoint	Status	Age,year	Gender,M(%)	T2DM,%	BMI,kg/m^2^	QualityScore
Pischon T	2005	Caucasian	US	prospective	P-B	CAD	cases	65.2±0.5	100	9.2	26.2±0.2	10
(HPFS study)							controls	65.1±0.4	100	4.4	25.7±0.2	
Pischon T	2005	Caucasian	US	prospective	P-B	CAD	cases	60.4±0.4	0	19.6	26.8±0.4	10
(NHS study)							controls	60.3±0.3	0	6.6	25.4±0.2	
Nassar BA	2006	Caucasian	Canada	retrospective	P-B	CAD	cases	45.5±4.0 (<50y)	74.5	0	^a^–	10
								73.7±5.4 (>65y)				
							controls	67±6.4	46	0	–	
Zee RY	2006	Caucasian	US	prospective	P-B	MI	cases	58.3±0.4	100	5.6	25.5±0.1	9
(PHS study)							controls	58.4±0.2	100	2.7	25±0.1	
Zafarmand MH	2008	Caucasian	Netherlands	Prospective	P-B	CAD	cases	60.5±5.9	0	5.7	26.8±3.9	10
(prospect-EPICstudy)							controls	57.1±6.1	0	2.2	25.8±4.0	
Dallongeville J	2009	Caucasian	Global	prospective	P-B	CAD	cases	61.5±7.9(M)	65.6	22.5	29.1±4.8(M)	9
(ADVANCEstudy)								60.0±8.8(F)			29.6±7.2(F)	
							controls	65.8±3.3(M)	53.8	10.7	28.3±4.4(M)	
								61.5±6.9(F)			27.4±6.3(F)	
Dallongeville J	2009	Caucasian	France&	prospective	P-B	CAD	cases	55.3±3.0	100	8.8	27.1±3.4	10
(PRIME study)			Northern Ireland				controls	55.1±2.8	100	4.9	26.7±3.5	
Evangelisti L	2009	Caucasian	Italy	retrospective	P-B	ACS	cases	66(25–89)	70.8	26.7	27(17–39.6)	11
							controls	64(20–89)	69.6	4.7	26(15.7–40.9)	
Vogel U	2009	Caucasian	Denmark	prospective	P-B	ACS	cases	58(51–65)(M)	76.2	5.4	26.9(22.4–34.1)(M)	11
(DHC study)								60(52–65)(F)			26.4(20.1–35.2)(F)	
							controls	56(51–64)(M)	53.2	1.7	26.3(21.6–32.4)(M)	
								56(51–64)(F)			24.6(19.7–33.7)(F)	
vos HL	2000	Caucasian	Netherlands	retrospective	H-B	MI	cases	–	100	–	–	9
							controls	–	100	–	–	
Bluher M	2002	Caucasian	Germany	retrospective	H-B	CAD	cases	67.1(43–91)	67.2	100	28.68(17.9–44.1)	12
							controls	63.3(33–87)	43.9	100	28.77(17.6–44.4)	
Tobin MD	2004	Caucasian	UK	retrospective	H-B	MI	cases	61.9±9.2	68	8.7	25.9±3.9	9
							controls	58.6±10.7	62	2	25.7±3.6	
Yilmaz-Aydogan H	2011	Caucasian	Turkey	retrospective	H-B	CAD	cases	59.22±11.96^b^	50.5	50.5	26.32±4.01^b^	10
								57.10±10.93^c^			26.55±3.15^c^	
							controls	57.03±12.65	55.2	–	25.33±3.54	
Shen D	2005	Asian	China	retrospective	H-B	CAD	cases	58.6±11.7	60.4	0	25.5±2.9	9
							controls	52.9±13.3	60.8	0	24.3±2.5	
Li L	2006	Asian	China	retrospective	H-B	MI	cases	64.95±10.79	73.4	–	24.21±3.54	8
							controls	62.1±8.23	55.3	–	24.57±3.32	
Rhee EJ	2007	Asian	Korea	retrospective	H-B	CAD	cases	58.8±9.8^d^	–	–	25.8±2.7^d^	10
								62.1±9.1^e^			25.8±2.9^e^	
								65.4±8.0^f^			24.7±2.9^f^	
							controls	55±11.5	–	–	25.3±2.9	
Wu SR	2007	Asian	China	retrospective	H-B	CAD	cases	69.55±10.58	65.1	–	–	10
							controls	64.76±11.93	57.1	–	–	
wang JJ	2008	Asian	China	retrospective	H-B	CAD	cases	–	–	59.2	–	8
							controls	–	–	45.2	–	
Wang YX	2009	Asian	China	retrospective	H-B	CAD	cases	63.8±7.4	58.1	100	25.1±3.2	8
							controls	53.7±10.4	54.2	100	24.9±3.8	
Ho JS	2012	Asian	China	prospective	H-B	CAD	cases	58(48.5–68)	43.5	100	24.9(23.2–26.9)	9
							controls	46(38–60)	40.3	100	24.7(22.3–27.3)	
Ruiz-Narvaez EA	2007	Others	Costa Rica	retrospective	P-B	MI	cases	58±11	74	25.5	26±4.1	8
							controls	58±11	74	14.5	26.5±4.2	
AshokKumar M	2010	Others	India	retrospective	P-B	CAD	cases	53.2±7.8	77.8	43.7	25.8±3.9	10
							controls	53.5±8.2	74.8	12.7	24.8±2.8	

P-B, population-based study; H-B, hospital-based study; M(%): male(percent); F: female; T2DM: type 2 diabetes mellitus; BMI: body mass index; ^a^: Data not available; ^b^: diabetes; ^c^: non-diabetes; ^d^: 1-stenotic vessel; ^e^: 2-stenotic vessels; ^f^: 3-stenotic vessels; Age and BMI are expressed as mean ± SD (standard deviation ) or median (5th and 95th percentiles ).

**Table 2:** The distribution of Pro12Ala genotypes and alleles among cases and controls, and P-values of HWE in controls.

First Author	sample size		Ala allele, %		Pro allele, %		AlaAla genotype		ProAla genotype		ProPro genotype		HWE,
	cases	controls	cases	controls	cases	controls	cases	controls	cases	controls	cases	controls	P value
AshokKumar M	414	424	8.7	7.3	91.3	92.7	5	4	62	54	347	366	0.21
Bluher M	201	164	7.7	7.9	92.3	92.1	4	2	23	22	174	140	0.30
Dallongeville J(ADVANCE study)	1076	805	12.0	12.2	88.0	87.8	12	9	231	174	816	605	0.37
Dallongeville J(PRIME study)	249	494	11.0	11.5	89.0	88.5	7	4	40	104	198	378	0.28
Evangelisti L	202	295	9.0	6.0	91.0	94.0	3	0	30	38	169	258	0.24
Ho JS	108	1309	0.9	2.7	99.1	97.3	0	0	2	71	105	1229	0.31
Li L	218	626	5.3	3.2	94.7	96.8	0	2	23	36	195	588	0.08
Nassar BA	300	150	10.3	12.0	89.7	88.0	0	0	62	36	238	114	0.09
Pischon T(NHS study)	245	485	12.7	10.8	87.3	89.2	4	6	54	93	187	386	0.88
Pischon T(HPFS study)	250	502	13.4	9.9	86.6	90.1	4	4	59	91	187	407	0.66
Rhee EJ	150	117	9.3	8.5	90.7	91.5	0	0	14	10	136	107	0.63
Ruiz-Narvaez EA	1805	1805	11.0	10.0	89.0	90.0	24	25	341	310	1440	1470	0.06
Shen D	96	125	6.2	3.6	93.8	96.4	1	1	10	7	85	117	0.14
Tobin MD	547	505	11.2	12.7	88.8	87.3	10	4	103	120	434	381	0.10
Vogel U	1031	1703	13.8	13.5	86.2	86.5	23	27	238	397	770	1245	0.47
vos HL	563	646	13.1	11.3	86.9	88.7	21	12	105	122	437	512	0.14
wang JJ	147	219	10.5	10.0	89.5	90.0	0	0	31	44	116	175	0.10
Wang YX	258	288	1.9	3.5	98.1	96.5	0	1	10	18	248	269	0.25
Wu SR	152	49	5.3	3.1	94.7	96.9	0	0	16	3	136	46	0.83
Yilmaz-Aydogan H	202	105	6.4	8.1	93.6	91.9	0	0	26	17	176	88	0.37
Zafarmand MH	211	1519	11.1	13.4	88.9	86.6	3	30	41	346	167	1143	0.52
Zee RY	523	2092	9.9	12.3	90.1	87.8	6	31	92	452	425	1611	0.91
Total	8948	14427	10.6	10.1	89.5	89.9	127	162	1613	2565	7187	11644	

HWE: Hardy–Weinberg equilibrium. The P-value of HWE determined by the χ^2^ test or Fisher's exact test in control groups.

**Figure 2:**
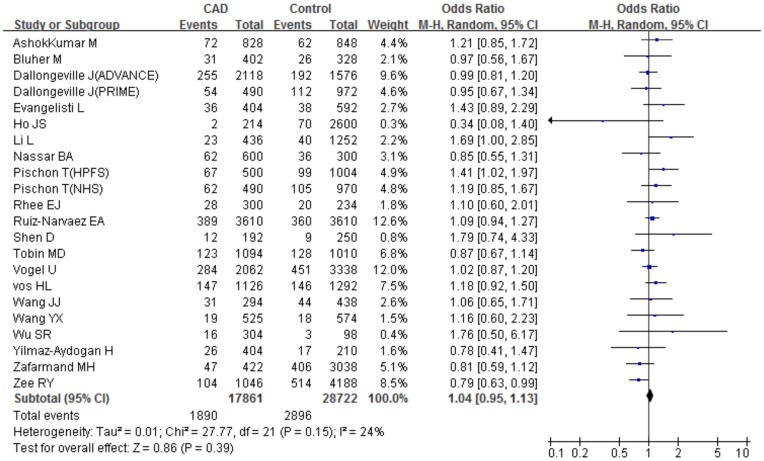
Meta-analysis for the overall association between the *PPARγ2* Pro12Ala polymorphism and CAD under the allele comparison (Ala vs Pro). ’Events’ indicates the total number of Ala allele. ‘Total’ indicates the total number of Pro allele plus Ala allele.

The main results of the meta-analysis and the heterogeneity test were presented in Table 3. For each study, we investigated the association between the *PPARγ2* Pro12Ala polymorphism and CAD risk under different genetic models. Overall, We did not detect any significant association under the allele comparison (Ala vs Pro: P = 0.39, OR = 1.04, 95%CI 0.95–1.13, P_heterogeneity_ = 0.15, I^2^ = 24%) and under the dominant genetic model with heterogeneity (ProAla+AlaAla vs ProPro: P = 0.88, OR = 1.01, 95%CI 0.91–1.11, P_heterogeneity_ = 0.06, I^2^ = 33%). However, a marginal increased risk of CAD under the recessive genetic model (AlaAla vs ProAla+ProPro: P = 0.04, OR = 1.31, 95%CI 1.01–1.69, P_heterogeneity_ = 0.67, I^2^ = 0%) and the homozygote comparison (AlaAla vs ProPro: P = 0.04, OR = 1.30, 95%CI 1.01–1.68, P_heterogeneity_ = 0.68, I^2^ = 0%) was observed for all the subjects (Figure 2).

**Table 3:** Summary estimates for ORs and 95% CI in different subgroups under various genetic contrasts.

Genotype contrasts	Study population	study number, (case/control), n(n/n)	P_heterogeneity_	I^2^,%	P value^a^	OR	95% CI
*Total studies*							
Allele comparison		22(8948/14427)	0.15	24	0.39	1.04	0.95–1.13
(Ala vs Pro)							
Dominant model		22(8948/14427)	0.06	33	0.88	1.01	0.91–1.11
(ProAla+AlaAla vs ProPro)							
Recessive model		16(7889/12478)	0.67	0	0.04	1.31	1.01–1.69
(AlaAla vs ProAla+ProPro)							
Homozygote comparison		16(7889/12478)	0.68	0	0.04	1.30	1.01–1.68
(ALaAla vs ProPro)							
*Ethnicity*							
Allele comparison	Asian	7(1129/2733)	0.41	3	0.11	1.24	0.96–1.61
	Caucasian	13(5600/9465)	0.13	31	0.94	1.00	0.90–1.10
	Others	2(2219/2229)	0.60	0	0.15	1.11	0.96–1.27
Dominant model	Asian	7(1129/2733)	0.10	43	0.56	1.13	0.75–1.69
	Caucasian	13(5600/9465)	0.18	26	0.44	0.96	0.86–1.07
	Others	2(2219/2229)	0.67	0	0.12	1.13	0.97–1.31
Recessive model	Asian	3(572/1039)	0.83	0	0.68	0.69	0.12–3.9
	Caucasian	11(5098/9210)	0.48	0	0.01	1.45	1.08–1.96
	Others	2(2219/2229)	0.69	0	0.99	1.00	0.60–1.69
Homozygote comparison	Asian	3(572/1039)	0.82	0	0.7	0.71	0.13–4.02
	Caucasian	11(5098/9210)	0.46	0	0.02	1.44	1.07–1.93
	Others	2(2219/2229)	0.69	0	0.92	1.03	0.61–1.72
*Study design*							
Allele comparison	prospective	8(3693/8909)	0.06	48	0.79	0.98	0.85–1.13
	retrospective	14(5255/5518)	0.55	0	0.07	1.09	0.99–1.20
Dominant model	prospective	8(3693/8909)	0.06	48	0.58	0.96	0.82–1.12
	retrospective	14(5255/5518)	0.25	18	0.39	1.06	0.93–1.20
Recessive model	prospective	7(3585/7600)	0.47	0	0.21	1.25	0.88–1.77
	retrospective	9(4304/4878)	0.61	0	0.09	1.39	0.95–2.01
Homozygote comparison	prospective	7(3585/7600)	0.44	0	0.24	1.23	0.87–1.74
	retrospective	9(4304/4878)	0.65	0	0.08	1.39	0.96–2.03
*Population source*							
Allele comparison	P-B	11(6306/10274)	0.10	38	0.64	1.03	0.93–1.14
	H-B	11(2642/4153)	0.33	12	0.39	1.07	0.92–1.26
Dominant model	P-B	11(6306/10274)	0.09	39	0.86	1.01	0.9–1.13
	H-B	11(2642/4153)	0.13	34	0.96	1.01	0.81–1.25
Recessive model	P-B	10(6006/10124)	0.51	0	0.24	1.19	0.89–1.59
	H-B	6(1883/2354)	0.87	0	0.03	1.85	1.07–3.19
Homozygote comparison	P-B	10(6006/10124)	0.49	0	0.24	1.19	0.89–1.58
	H-B	6(1883/2354)	0.88	0	0.03	1.83	1.06–3.16
*Endpoint*							
Allele comparison	CAD	15(4059/6755)	0.46	0	0.5	1.04	0.93–1.15
	ACS	2(1233/1998)	0.19	41	0.45	1.12	0.84–1.49
	MI	5(3656/5674)	0.02	67	0.78	1.03	0.85–1.25
Dominant model	CAD	15(4059/6755)	0.27	17	0.97	1.00	0.88–1.15
	ACS	2(1233/1998)	0.29	9	0.71	1.04	0.85–1.27
	MI	5(3656/5674)	0.01	70	0.93	1.01	0.81–1.26
Recessive model	CAD	9(3000/4806)	0.77	0	0.23	1.32	0.84–2.07
	ACS	2(1233/1998)	0.19	43	0.35	2.22	0.41–11.91
	MI	5(3656/5674)	0.27	22	0.33	1.25	0.80–1.97
Homozygote comparison	CAD	9(3000/4806)	0.77	0	0.23	1.32	0.84–2.07
	ACS	2(1233/1998)	0.18	44	0.36	2.26	0.40–12.74
	MI	5(3656/5674)	0.28	21	0.35	1.24	0.79–1.94

a:Test for overall effect;P-B: population-based, H-B: hospital-based.

### Subgroup Analysis

**Figure 3:**
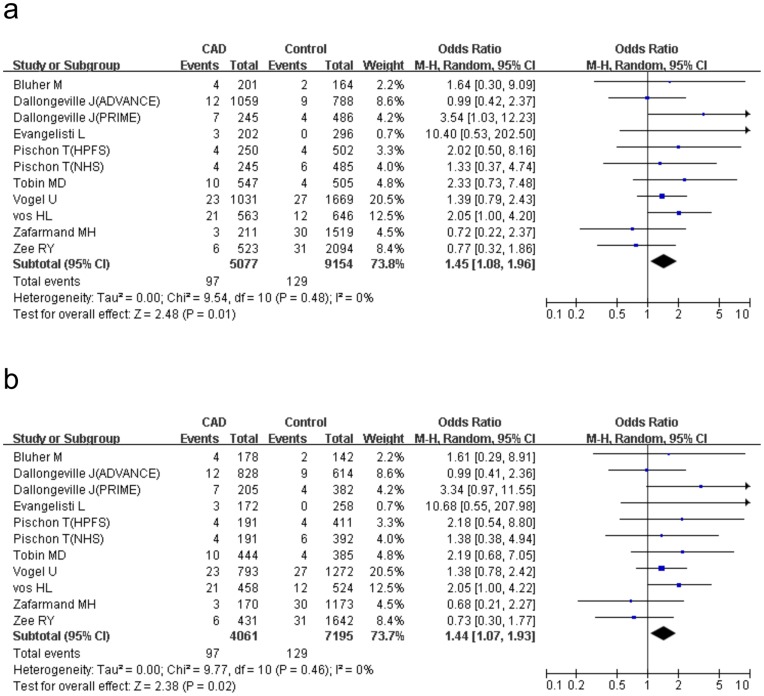
Meta-analysis for the association between *PPARγ2* Pro12Ala polymorphism and CAD among Caucasians. The AlaAla homozygote shows a significant increased risk of CAD under the recessive model (AlaAla vs ProAla+ ProPro, Figure 3a) and under the homozygote comparison (AlaAla vs ProPro, Figure 3b). ‘Events’ indicates the total number of AlaAla genotype. ‘Total’ indicates the total number of AlaAla genotype plus ProAla+ ProPro genotype (Figure 3a) and the total number of AlaAla genotype plus ProPro genotype (Figure 3b) respectively.

We conducted a series of subgroup analysis on ethnicity, study design, population source and endpoints to explore the potential causes of the heterogeneity (Table 3). Data of all the 22 studies were stratified according to the 3 different ethnic groups: Asian (7 studies involved 1129 cases and 2733 controls), Caucasian (13 studies involved 5600 cases and 9465 controls) and others (1 study recruited Costa Rican and the other recruited Indian). The “others” group contained 2219 cases and 2229 controls. Non-significant association was observed in all ethnic subgroups under the allele comparison and the dominant genetic model. Nevertheless, the significance of the increased CAD risk was augmented among Caucasians under the recessive model (P = 0.01, OR = 1.45, 95%CI 1.08–1.96, P_heterogeneity_ = 0.48, I^2^ = 0%) and the homozygote comparison (P = 0.02, OR = 1.44, 95%CI 1.07–1.93, P_heterogeneity_ = 0.46, I^2^ = 0%) compared with the overall estimation (Figure 3). In contrast, there was non-significant changes in CAD risk among Asians (recessive model:P = 0.68, OR = 0.69, 95%CI 0.12–3.90, P_heterogeneity_ = 0.83, I^2^ = 0% ; homozygote comparison:P = 0.70,OR = 0.71, 95%CI 0.13–4.02, P_heterogeneity_ = 0.82, I^2^ = 0%) and mixed-blood population (recessive model:P = 0.99, OR = 1.00, 95%CI 0.60–1.69, P_heterogeneity_ = 0.69, I^2^ = 0%;homozygote comparison:P = 0.92,OR = 1.03, 95%CI 0.61–1.72, P_heterogeneity_ = 0.69, I^2^ = 0%).

**Figure 4:**
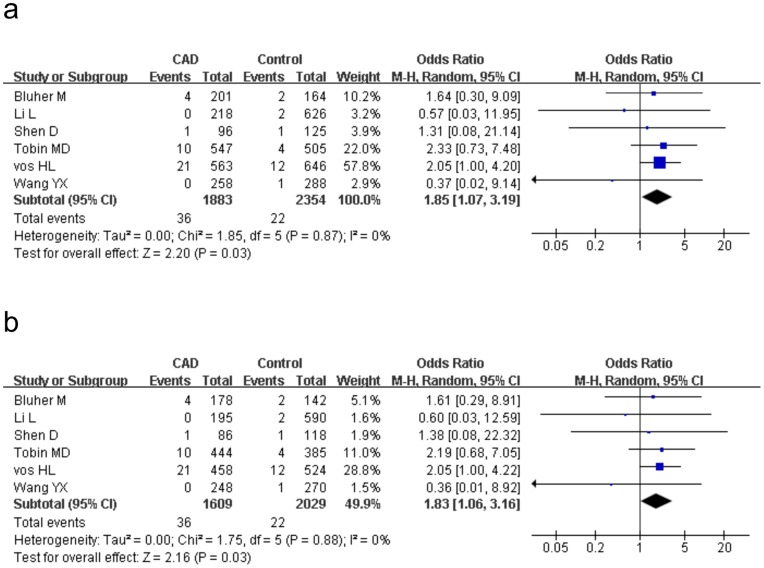
Meta-analysis for the association between *PPARγ2* Pro12Ala polymorphism and CAD in hospital-based studies. The Pro12Ala polymorphism shows a signification increased risk of CAD under the recessive model (AlaAla vs ProAla+ ProPro, Figure 4a) and under the homozygote comparison (AlaAla vs ProPro, Figure 4b). ‘Events’ indicates the total number of AlaAla genotype. ‘Total’ indicates the total number of AlaAla genotype plus ProAla+ ProPro genotype (Figure 4a) and the total number of AlaAla genotype plus ProPro genotype (Figure 4b) respectively.

A further subgroup analysis was performed in light of study design. 11 studies included 2642 cases and 4113 controls were H–B and the other half involved 6306 cases and 10274 controls were P-B. After dividing into population source, the CAD risk magnitude of H-B studies was distinctly strengthened under the recessive model (P = 0.03, OR = 1.85, 95%CI 1.07–3.19, P_heterogeneity_ = 0.87, I^2^ = 0%) and the homozygote comparison (P = 0.03,OR = 1.83, 95%CI 1.06–3.16, P_heterogeneity_ = 0.88, I^2^ = 0%) (Figure 4), whereas in P-B subjects, the lack of remarkable association was found between the *PPARγ2* Pro12Ala polymorphism and CAD under all genetic models (allele comparison: P = 0.64, OR = 1.03, 95%CI 0.93–1.14, P_heterogeneity_ = 0.10, I^2^ = 38%). Further analysis stratifying on study design (prospective versus retrospective) or endpoints (CAD versus ACS versus MI) yielded no significant association under the four genetic models in all the subgroups.

### Sensitivity Analysis

Sensitivity analysis was performed to estimate the heterogeneity among all the studies in our meta-analysis. We sequentially removed the single study every time to ascertain the cause of heterogeneity. As a result, 2 independent studies (Zee *et al.* [44] and Pischon [HPFS] *et al.* [22]) accounted for the major sources of heterogeneity. The overall heterogeneity of the Pro12Ala polymorphism no longer existed when these 2 studies were ruled out respectively in the total analysis under the four genetic models (P_heterogeneity_>0.10) and the total effect estimation remained negative. Meanwhile, similar results was also observed in the subsequent subgroup analysis (P_heterogeneity_>0.10). Nevertheless, there was not any single study influencing the pooled ORs significantly in any subgroups.

### Cumulative Analysis

There was no remarkable evidence suggesting that the first published study had significant impact on the subsequent publication by the cumulative meta-analysis (data not shown).

### Meta-regression Analysis

**Figure 5:**
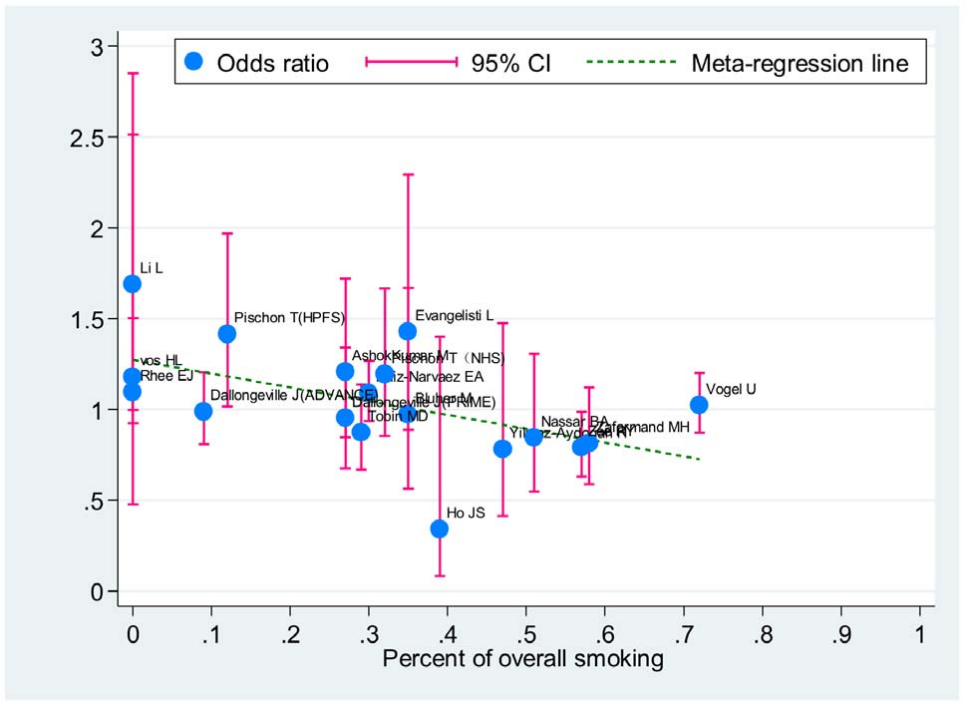
Meta-regression of overall smoking percent on in-allele risk estimates of *PPARγ2* Pro12Ala polymorphism. For each study, OR is shown by the middle of the blue solid circle whose upper and lower extremes represent the corresponding 95%CI. OR values were calculated for the smokers against non-smokers when available. The green dotted line is plotted by fitting OR and overall smoking percent for the included studies.

The meta-regression was a feasible scenario to identify the further source of heterogeneity. The mean or median value of age and BMI and the proportion of male, smoking and T2DM were involved in the meta-regression. Among these variable, the association of Pro12Ala polymorphism with CAD risk was shown with a low smoking rate under the allele comparison (correlation coefficient:−0.53, P = 0.02) (Figure 5).

### Publication Bias

**Figure 6:**
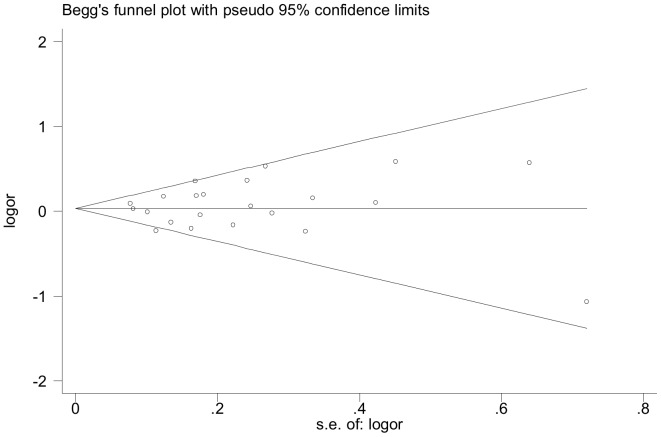
Begg’s funnel plot analysis to detect publication bias for allele comparison (Ala vs Pro) of the Pro12Ala polymorphism.

The funnel plot was applied for allele comparison in the OR analysis of the *PPARγ2* Pro12Ala polymorphism to evaluate the publication bias of the literatures. There was no evidence for remarkable publication bias of the Pro12Ala polymorphism (t = −0.12, P = 0.91 for Ala vs Pro) confirmed by the Egger’s test and Begg’s funnel plot (Figure 6).

## Discussion

The definite association of *PPARγ2* Pro12Ala polymorphism with CAD risk is not precisely clarified yet. Although some studies have been performed to connect *PPARγ2* Pro12Ala polymorphism with CAD, the results remained conflicting. The varying results may be explained by a relatively small size of samples and the limited statistical power of single studies but may also be explained by different distributions of potential effect modifiers in the study populations. To our knowledge, this is one of the largest systematic reviews of the literatures via a meta-analysis to investigate the relationship between the *PPARγ2* common polymorphism and the potential risk of CAD. Overall results verified that the AlaAla homozygote of *PPARγ2* Pro12Ala polymorphism might have marginal significant increased risk of CAD in a recessive inherited pattern. Our result is subtly different from a previous meta-analysis containing 6898 CAD cases and 11287 controls by Dallongeville *et al.* [36], which revealed that the *PPAPγ2* Pro12Ala polymorphism had a borderline non-significant increased risk of CAD (P = 0.06, OR = 1.29, 95%CI 0.99–1.67) under the recessive genetic model. The nuance is probably due to the restricted sample size.

Genetic heterogeneity is inevitable in disease identification strategy [55] and subgroup analysis determined ethnicity as a potential cause of between-study difference. We found that the association of the *PPARγ2* Pro12Ala polymorphism with CAD risk was different between Caucasians and other ethnic groups. The AlaAla genotype carriers showed a significant 45% risk increase among Caucasians, whereas the significance was lack among Asians and other mixed-blood population. Our result indicated the Pro12Ala polymorphism might be increased risk conferring locus for CAD in Caucasian population, but not in Asian and other population. The consensus has not been reached, suggesting the racial genetic diversity of the PPARγ2 Pro12Ala polymorphism plays an important role in the etiology of atherosclerosis across various ethnic populations. It also should be noticed that the Pro12Ala polymorphism was hypervariable between different ancestries [56] and might have subtle influences on the result of case-control studies.

Apart from the dramatic impact of ethnicity on total evaluation, another estimate should be treated with caution when studies were stratified by population source. Risk increase given by the AlaAla homozygote in the H-B studies seemed to differ from that in P-B studies, being 85% in H-B studies and 19% in P-B studies under the recessive model. Risk increase was significant in H-B studies but not in P-B studies. Besides the relatively small sample size, population classification was still problematic [57]. Despite high participation and less information bias may favor H-B studies, the weakness of H-B studies is ineluctable. Hospital controls are derived from different source population and partly represent the general population in the study region. In addition, the possibility of biased case-control comparisons should be taken into account when controls were selected from a ill-related study base [43] and could not accurately reflect the exposure experience of the real source population. By contrast, the controls sampled from community or general population are largely regarded as being more advisable than those from hospital for reasons of representativeness. Considering a wide range of confidence intervals of in the H-B subgroup analysis, further studies are called for to ascertain the reliability of effect size.

Furthermore, our meta-regression analysis found out a link of the *PPARγ2* Pro12Ala polymorphism with CAD risk in population with lower smoking proportion. Considering smoking is a major risk factor of CAD [58], our result implied potential interaction of the Pro12Ala genotype with environment factors.

Although our meta-analysis included relatively large sample size consistent of HWE, there are some methodological limitations should be noticed [59]. The literature bias is a latent issue. Because small negative studies are less likely to be accepted to publish and the articles in languages other than English and Chinese were excluded, the possibility of language bias cannot be ruled out completely, even though the Egger’s test and funnel plots did not provide any evidence of publication bias in our meta-analysis. Although simulation studies of funnel plots have documented publication bias may be inferred by mistake if heterogeneity of the studies is present [60], there is still no gold standard against the methods to compare the results of funnel plot tests and Egger’s test [61].

A majority of in vivo studies showed PPARγ2 exerts direct and indirect anti-inflammatory effects in the arterial cells of the vascular wall. PPARγ2 activation reduces the production of macrophage and lymphocyte cytokine, inhibits the growth, proliferation [62], [63], and migration of vascular smooth muscle cell as well as restrains the expression of endothelial cell adhesion molecule, chemokine, and matrix metalloproteinase [64]. All of these evidences suggested that PPARγ2 is benefit to prevent the initiation of atherosclerosis [65]. Nevertheless, clinical studies determining the role of the *PPARγ2* Pro12Ala polymorphism in CAD are scarce. Our meta-analysis complements the evidences that the Pro12Ala polymorphism, a loss of PPARγ2 function mutation, may exert pleiotropic and deleterious effects in the development of atherosclerosis.

In conclusion,our meta-analysis, comprising 23375 participants,implies that homozygosity of the Ala allele might have a potential increased risk of CAD. The effect is at odds, being stronger in Caucasians and barely significant in Asians. Our meta-analysis also emphasizes the necessity of great caution when trying to interpret and reconcile data observed in different ethnic population. More prospective registered studies are helpful to confirm or refuse the present association.

## Supporting Information

Table S1 **Criteria of quality assessment for genetic association of the PPARγ2 gene Pro12Ala polymorphism with CAD.**
(DOC)

Checklist S1 **PRISMA Checklist.**
(DOC)
