# Supplementary material for: The Pro12Ala Polymorphism in the Peroxisome Proliferator-Activated Receptor Gamma-2 Gene (PPARγ2) Is Associated with Increased Risk of Coronary Artery Disease: A Meta-Analysis
Source: PLoS One. 2012 Dec 31;7(12):e53105. doi: 10.1371/journal.pone.0053105 (PMC3534032; doi:10.1371/journal.pone.0053105)
Supplement: Table S1 — Criteria of quality assessment for genetic association of the PPARγ2 gene Pro12Ala polymorphism with CAD. (DOC) [file pone.0053105.s001.doc]

| TableS1 Criteria of quality assessment for genetic association of the *PPARγ2* gene Pro12Ala polymorphism with CAD | |
| --- | --- |
| **Criteria** | **Quality score** |
| *i.Representativeness of case-patients* |  |
| Consecutive/randomly selected from case population with clearly defined sampling frame | 2 |
| Consecutive/randomly selected from case population without clearly defined random frame or with extensive inclusion criteria | 1 |
| Method of selection not described | 0 |
|  |  |
| *ii.Representativeness of controls* |  |
| Controls were consecutive/randomly drawn from the same sampling frame (ward/community) as cases with the same criteria | 2 |
| Controls were consecutive/randomly drawn from a different area than cases | 1 |
| Method of selection not described | 0 |
|  |  |
| *iii.Ascertainment of CAD* |  |
| Clearly described objective criteria for diagnosis of CAD | 2 |
| Diagnosis of CAD by patient self-report or by patient history | 1 |
| Not described | 0 |
|  |  |
| *iv.Ascertainment of controls* |  |
| Clinical examinations were performed on controls to prove that controls did not have CAD | 2 |
| Article merely stated that controls were subjects who did not report CAD, no evidence provided | 1 |
| Not described | 0 |
|  |  |
| *v.Ascertainment of genotyping examination* |  |
| Genotyping performed under “blind” conditions | 1 |
| Unblinded or not mentioned | 0 |
|  |  |
| *vi.Test for Hardy-Weinberg equilibrium* |  |
| Hardy-Weinberg equilibrium in control group | 2 |
| Hardy-Weinberg disequilibrium in control group | 1 |
| Hardy-Weinberg equilibrium not checked | 0 |
|  |  |
| *vii.Association assessment* |  |
| Assessed association between genotypes and CAD with appropriate statistics and adjustment for confounders | 2 |
| Assessed association between genotypes and CAD with appropriate statistic without adjustment for confounders | 1 |
| Inappropriate statistics used | 0 |
|  |  |
| *Total* | 13 |
|  |  |
